# CD14 C-159T and Toll-Like Receptor 4 Asp299Gly Polymorphisms in Surviving Meningococcal Disease Patients

**DOI:** 10.1371/journal.pone.0007374

**Published:** 2009-10-07

**Authors:** Ariane Biebl, Axel Muendlein, Zhyrgal Kazakbaeva, Sigrid Heuberger, Gudrun Sonderegger, Heinz Drexel, Susanne Lau, Renate Nickel, Michael Kabesch, Burkhard Simma

**Affiliations:** 1 Department of Pediatrics, University Teaching Hospital, Feldkirch, Austria; 2 Vorarlberg Institute for Vascular Investigation and Treatment (VIVIT), Academic Teaching Hospital, Feldkirch, Austria; 3 National Reference Center for Meningococcal Disease, Graz, Austria; 4 University of Human Sciences of the Principality of Liechtenstein, Triesen, Principality of Liechtenstein; 5 Department of Pediatrics, University Hospital of Berlin, ChariteÌ, Germany; 6 Department of Pulmonology and Allergy, University Hospital of Hannover, Hannover, Germany; LMU University of Munich, Germany

## Abstract

**Background:**

Carriage of Neisseria meningitidis occurs approximately in 10% of the population, onset of invasive meningococcal disease (IMD) cannot be predicted and differs between ages. It remains unclear, which host factors determine invasion of the bloodstream by the bacteria. Innate immunity has a very important role in the first recognition of invading pathogens. The functional single nucleotide polymorphisms (SNPs) CD14 C-159T and toll-like receptor 4 (TLR4) Asp299Gly have been associated with the risk of gram-negative infections. However, their role in development of IMD still remains unclear. Our aim was to investigate the influence of CD14 C-159T and TLR4 Asp299Gly polymorphisms on the risk of IMD.

**Methodology/Principal Findings:**

It was a retrospective case control study. Surviving Austrian meningococcal disease patients were enrolled by sending buccal swabs for DNA analysis. 185 cases with a proven meningococcal infection and 770 healthy controls were enrolled. In surviving meningococcal disease patients DNA analysis of CD14 C-159T and TLR 4 Asp299Gly polymorphisms was performed, as they are part of the innate immune response to bacterial determinants. CD14 C-159T and TLR4 Asp299Gly SNPs were not significantly associated with the presence of IMD when compared to healthy controls. The odds ratio for CD14 C-159T SNP was 1.14 (95% confidence interval (CI) 0.91–1.43; p = 0.266). In TLR4 Asp 299 Gly SNP the odds ratio was 0.78 (CI 0.47–1.43; p = 0.359).

**Conclusion/Significance:**

We could not observe a significant influence of CD14 C-159T and TLR4 Asp299Gly polymorphisms on the risk of developing IMD in surviving meningococcal disease patients. To our knowledge, this is the first study investigating the influence of the CD14 C-159T SNP on the susceptibility to IMD.

## Introduction

In the industrialized world invasive meningococcal disease (IMD) remains a major cause of death and disability in children and adolescent. IMD is a rare but dangerous infection with the gram- negative bacterium *Neisseria meningitides (N.meningitidis)*. The disease is most common in young infants and children with a smaller, secondary peak in adolescents. In contrast, carriage of *N. meningitidis* is highest in adolescents (up to 30% university campus e.g.) and lowest in young children (2–5%) [1]–[3]. Carriage of *N. meningitidis* is mostly asymptomatic, especially in adolescents. The understanding of the transmission process from carriage to disease with *N.meningitidis* is crucial for the development of effective disease control mechanism and to define a special patient risk profile.

Successful defence against N. *meningitidis* infection relies on an innate immune response to bacterial determinants, such as lipopolysaccharides (LPS) [4]. Cluster of differentiation (CD) 14 is a pattern recognition receptor playing an essential role in innate immunity [5]. CD14 is found on the membrane of monocytes and as a soluble form in serum [6]–[7]. Along with LPS-binding protein, CD14 acts to transfer LPS (and other bacterial ligands) to the Toll-like receptor 4 (TLR4)/MD-2 signalling complex. This results in the activation of inflammatory gene expression.

Recent research indicates that there is significant individual diversity in CD14/TLR4 mediated response. Therefore, a single nucleotide polymorphism (SNP) in the TLR4 gene resulting in an Asp to Gly substitution at codon 299 alters the extracellular domain of the receptor, and airway epithelial cells of patients carrying this polymorphism are hyporesponsive to LPS [8].

A polymorphism in the CD14 gene leads to a C-to-T change at position −159 in the proximal promoter region and was found to be associated with increased CD14 expression [9]. Homozygous carriers of the T allele have significant increases in both soluble and membrane-bound CD14 [10]–[11]. Increased CD14 levels are associated with inflammatory infectious diseases and poor outcome in Gram-negative sepsis [12]. The variant may therefore influence host recognition and clearance of bacteria. The impact of CD14 C-159T SNP on the risk of gram-negative infections has been investigated in several other studies [13]–[16]. However, the influence of CD14 C-159T SNP on the risk of infection with *N. meningitidis* and development of IMD has not been studied yet. Only few reports exist investigating the relation between TLR4 Asp299Gly SNP and the susceptibility to IMD [17]–[18], but results remain controversial.

Therefore, we conducted a case-control study to elucidate the association of CD14 C-159T and TLR4 Asp299Gly SNPs on the susceptibility to IMD. We wanted to know, if a genetically determined deficiency in sensing meningococcal LPS increases risk of disease.

## Methods

### Patients

The study was conducted as retrospective case-control study involving 185 Caucasian patients (children, adolescent, and adults) with confirmed diagnosis of IMD and 770 healthy controls. Patients were notified to the Federal Office of Public Health or the National Reference Centre for IMD in Austria during 1993 to 2004. IMD was defined as culture or detection by PCR of *N. meningitidis* from blood or cerebrospinal fluid. We excluded patients with clinical signs and symptoms of acute meningococcal infection but negative cultures. Patients were invited by mail to participate in the study. Cells for DNA-preparation were collected directly by the patients using buccal swabs. Clinical, demographic and laboratory data were collected in collaboration with the National Reference Center for IMD in Austria. Age at disease onset, gender, clinical picture of meningitis, sepsis or both, Glasgow Outcome Scale and serotypes were recorded. DNA from 770 Caucasians, who participated as controls in the Multicentre Allergy Study (MAS study), were used as reference controls [19]. The study was approved by the ethics committee of the Medical University of Innsbruck and written informed consent was obtained from each patient.

### Genotyping

Genomic DNA was extracted from buccal swabs by standardized techniques using the peqGOLD® Blood DNA Mini kit (PEQLAB Biotechnologie Ltd., Erlangen, Germany). Reference samples were previously genotyped for the CD14 C-159T SNP [20] and were re-genotyped to avoid any methodical bias. Genotyping was carried out by the 5′ nuclease assay using TaqMan® MGB probes on an ABI Prism® 7000 Sequence Detection System (Applied Biosystems, Forster City, CA). TaqMan® MGB probes were provided together with corresponding PCR primers by the Assay-on-demand™ service (Applied Biosystems). Positive controls as well as non template controls were included in each run. Genotypes were determined by SDS (version 1.1) software followed by a visual control of accurate genotype classification.

### Statistical analyses

The Chi-squared test and univariate logistic regression analysis were used to evaluate the association between CD14 C-159T , TLR4 Asp299Gly and IMD. Statistical significance was defined as a two-tailed p value <0.05. Determined genotype frequencies were tested for Hardy-Weinberg equilibrium by chi-square testing with one degree of freedom. Statistical analyses were performed with the software package SPSS 11.0 for Windows (SPSS, Inc., Chicago, IL, USA).

Statistical power analysis was performed using the PS program (Dupont and Plummer, Jr., 1990). On the basis of published frequency of the putative risk genotypes, i.e. homozygous for the CD14 -159T allele (10) and heterozygous or homozygous for the TLR4 299Gly allele (17), respectively, *a priori* power analysis indicated that our study including 185 IMD cases and 770 controls can provide a statistical power of 80% to demonstrate an odds ratio (OR) of 1.68 and 1.88, respectively, for the association between the risk genotypes and IMD at an alpha fault of 0.05.

## Results

### Patients

**Figure 1:**
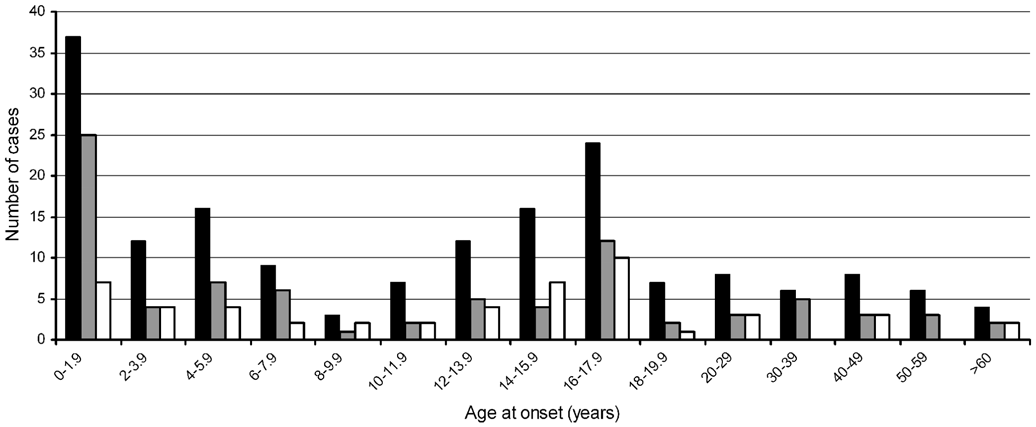
Meningococcal disease, age of infection and serogroup distribution. Numbers of cases are plotted against age of infection, presented as age groups in a 2-year interval, with respect to serogroups. Black bars indicate all cases, grey bars indicate serogroup B, and white bars indicate serogroup C.

Patients and controls showed a similar gender distribution (52.2% and 52.6% males within cases and controls, respectively). Age at onset of IMD was widely scattered, ranging from 0.1 to 74 years (Figure 1). Disease rate was highest in infants (below age of two years) and declined during childhood. The number of cases increased again in adolescents with a second peak in the age of 16 to 18 years. In adults (age>18 years) the prevalence of disease was lower and rather stable. Clinical characteristics of patients were as follows. State of health and immunocompetence were good in most of the patients, comorbidity was rare, as this were mainly pediatric patients. Most of the time meningococcal disease occurs in previously healthy persons. Gender was equally distributed between patients and controls. In surviving meningococcal disease patients serogroup B was the most frequent serogroup (60.4%) followed by serogroup C ( 36.7%) and others. The figure shows number of patients and distribution of serogroup B and C in 2-year interval. In young children serogroup B predominates, in contrast serogroupB and C are almost equal in adolescent and young adults. Sepsis (47%) was more often seen in patients compared to meningitis (31.5%) or mixed clinical picture (21.5%). The majority (78.3%) of our surviving meningococcal disease patients had a Glasgow outcome scale (GOS) of 5 (complete recovery), 15.1% had minor disability (minor hearing loss), 6.6% displayed major disability (amputation of limbs, major hearing or visual loss).

### DNA analysis

Genotype frequencies of CD14 C-159T and TLR 4 Asp299Gly are presented in table 1 and 2. All determined genotype frequencies were under Hardy-Weinberg equilibrium. Table 1 presents data on genotype distribution of the CD14 C-159T and TLR Asp299Gly SNP's among patients with IMD and the control group. Distribution did not significantly differ among IMD patients and controls. To further address any associations between SNPs and IMD we used different genetic models (dominant, additive, and, if appropriate, recessive) (table 2). Neither CD14 C-159T (OR 1.14; 95% CI 0.91–1.43; p = 0.266) nor TLR4 Asp299Gly SNP (OR 0.78; 95% CI 0.47–1.43; p = 0.359) was significantly associated with the susceptibility to IMD in any genetic model.

**Table 1:** Genotype distribution of the CD14 C-159T and TLR4 Asp299Gly variants among patients with meningococcal disease compared to controls.

		Genotype	
SNP		1/1	1/2	2/2	p value
CD14 C-159T	Patients	46 (24.9)	93 (50.3)	46 (24.9)	0.266
	Controls	213 (27.7)	393 (51.0)	164 (21.3)	
TLR4 Asp299Gly	Patients	167 (90.3)	18 (9.7)	0 (0.0)	0.359
	Controls	678 (88.2)	88 (11.4)	3 (0.4)	

Data are numbers of subjects. Numbers in parentheses present percent values. The frequent allele is denoted as “1” and the rare allele as “2”. P values were calculated with the Chi-squared test.

**Table 2:** Associations between CD14 C-159T and TLR4 Asp299Gly variants with IMD using different genetic models.

SNP	Model	OR	95% CI	P
	Dominant	1.16	0.80–1.67	0.442
CD14 C-159T	Recessive	1.22	0.84–1.78	0.294
	Additive	1.14	0.91–1.43	0.266
TLR4 Asp299Gly	Dominant	0.84	0.49–1.46	0.545
	Additive	0.78	0.47–1.43	0.359

Odds ratios (OR) and 95% confidence intervals (CI) were obtained from univariate logistic regression analysis. Due to minor allele frequency of the 299Gly allele of TLR4, no recessive genetic model was applied.

## Discussion

This is the first time that data on CD14 C-159T SNP on the risk of IMD are presented. Overall, CD14 C-159T and TLR4 Asp299Gly SNP were not significantly associated with the prevalence of IMD in survivors. Data about genetic influence on IMD are rare and controversial [21]–[22]. Our study presents data in a large patient and control cohort.

The impact of the CD14 C-159T SNP on the risk of gram-negative infection has been examined in several studies providing controversial results [13]–[16]. Sutherland provided data on CD14 and TLR 2 in patients with systemic inflammatory response syndrome [16]. Rupp et al investigated CD14 SNP with susceptibility to chronic Chlamydia pneumoniae infection [15], and Agnese and Jessen found no association with CD14 SNP and gram-negative infection [13], [14]. However, no data have been published investigating the influence of the CD14 C-159T SNP on the susceptibility to IMD. This was unexpected, as CD14 plays an essential role in innate immune response to bacterial lipopolysaccharides (LPS) [4].

Only few reports exist investigating the relation between TLR4 Asp299Gly and susceptibility to IMD (17–18). Faber et al [17] reported, that allele frequency was not higher in the overall patient population (197 patients versus 214 controls), but a significantly higher frequency was seen in 40 patients younger than 12 months of age. As TLR4 is part of the innate immune response and a mutation in this gene alters the extracellular domain of the receptor, one would expect a higher susceptibility to IMD, if this mutation is present. However, this was not seen in our patient cohort with 185 IMD survivors when compared to 770 healthy controls. This is also supported by Read et al [18], who also found no association with susceptibility and severity of IMD in 1047 IMD patients when compared to 879 controls.

Meningococcal infection is a disease with a wide range of clinical presentations and outcomes. Mortality is still high. With such a clinical profile, it is important to elucidate genetic determinants that may assist to attenuate its incidence or severity. As reported earlier data on TLR4 Asp299Gly remain controversial, our study gives negative results in a rather large patient and control group.

Some limitations of our study should be reported. Since we have chosen a retrospective case-control study design, we could not collect genetic data of patients, who died from IMD, probably leading to a certain genetic bias. It might well be that IMD is a multifactorial event and the selected group of study participants had survived because there was no difference to the control group. With respect to this possibility, CD14 and TLR4 polymorphisms could even have a positive effect on case fatality rate of IMD. In contrast, Read and al [18] found no association with TLR4 sequence variant and severity of IMD in a large study cohort, as mentioned above. Therefore, any genetic effects on disease severity remain debatable. It might well be that other single nucleotide polymorphisms could play a significant role in the susceptibility to IMD. However, we have chosen SNP CD14 C-159T and TLR 4 Asp299Gly, as i) functional roles of these SNPs have been described, altering gene expression and protein function, respectively [8]–[11], and ii) the respective genes act together and play important roles in innate immunity [5]–[7]. Nevertheless, we cannot exclude that other, probably rare sequence variants in CD14 and TLR4 may exert causal effects on the development of IMD, as this was previously described by others [23].

We conclude that CD14 C-159T and TLR 4 Asp299Gly SNP's were not significantly associated with the prevalence in IMD survivors. To our knowledge, this is the first study investigating the influence of the CD14 C-159T SNP on the susceptibility to IMD. Data are now required to confirm our findings.
